# Rapid Karyotype Evolution in *Lasiopodomys* Involved at Least Two Autosome – Sex Chromosome Translocations

**DOI:** 10.1371/journal.pone.0167653

**Published:** 2016-12-09

**Authors:** Olga L. Gladkikh, Svetlana A. Romanenko, Natalya A. Lemskaya, Natalya A. Serdyukova, Patricia C. M. O’Brien, Julia M. Kovalskaya, Antonina V. Smorkatcheva, Feodor N. Golenishchev, Polina L. Perelman, Vladimir A. Trifonov, Malcolm A. Ferguson-Smith, Fengtang Yang, Alexander S. Graphodatsky

**Affiliations:** 1 Institute of Molecular and Cellular Biology, Siberian Branch of the Russian Academy of Sciences, Novosibirsk, Russia; 2 Novosibirsk State University, Novosibirsk, Russia; 3 Cambridge Resource Centre for Comparative Genomics, Department of Veterinary Medicine, University of Cambridge, Cambridge, United Kingdom; 4 Severtzov Institute of Ecology and Evolution, Russian Academy of Sciences, Moscow, Russia; 5 Department of Vertebrate Zoology, Saint Petersburg State University, Saint Petersburg, Russia; 6 Zoological Institute, Russian Academy of Sciences, Saint Petersburg, Russia; 7 Wellcome Trust Sanger Institute, Wellcome Genome Campus, Hinxton, Cambridge, United Kingdom; Virginia Tech, UNITED STATES

## Abstract

The generic status of *Lasiopodomys* and its division into subgenera *Lasiopodomys* (*L*. *mandarinus*, *L*. *brandtii*) and *Stenocranius* (*L*. *gregalis*, *L*. *raddei*) are not generally accepted because of contradictions between the morphological and molecular data. To obtain cytogenetic evidence for the *Lasiopodomys* genus and its subgenera and to test the autosome to sex chromosome translocation hypothesis of sex chromosome complex origin in *L*. *mandarinus* proposed previously, we hybridized chromosome painting probes from the field vole (*Microtus agrestis*, MAG) and the Arctic lemming (*Dicrostonyx torquatus*, DTO) onto the metaphases of a female Mandarin vole (*L*. *mandarinus*, 2n = 47) and a male Brandt's vole (*L*. *brandtii*, 2n = 34). In addition, we hybridized Arctic lemming painting probes onto chromosomes of a female narrow-headed vole (*L*. *gregalis*, 2n = 36). Cross-species painting revealed three cytogenetic signatures (MAG12/18, 17a/19, and 22/24) that could validate the genus *Lasiopodomys* and indicate the evolutionary affinity of *L*. *gregalis* to the genus. Moreover, all three species retained the associations MAG1bc/17b and 2/8a detected previously in karyotypes of all arvicolins studied. The associations MAG2a/8a/19b, 8b/21, 9b/23, 11/13b, 12b/18, 17a/19a, and 5 fissions of ancestral segments appear to be characteristic for the subgenus *Lasiopodomys*. We also validated the autosome to sex chromosome translocation hypothesis on the origin of complex sex chromosomes in *L*. *mandarinus*. Two translocations of autosomes onto the ancestral X chromosome in *L*. *mandarinus* led to a complex of neo-X_1_, neo-X_2_, and neo-X_3_ elements. Our results demonstrate that genus *Lasiopodomys* represents a striking example of rapid chromosome evolution involving both autosomes and sex chromosomes. Multiple reshuffling events including Robertsonian fusions, chromosomal fissions, inversions and heterochromatin expansion have led to the formation of modern species karyotypes in a very short time, about 2.4 MY.

## Introduction

Rodentia is the most species-rich mammalian order and includes several important laboratory model species. The tribe Arvicolini is particularly notable for its extensive phenotypic and chromosomal variations which raised many questions about species dispersal and genomic processes accompanying speciation.

The generic status of *Lasiopodomys* Lataste, 1887 from the subfamily Arvicolinae (Cricetidae, Rodentia) is not universally accepted because of contradictions between the morphological and molecular data. Until recently, the genus *Lasiopodomys* included *L*. *mandarinus* Milne-Edwards, 1871, *L*. *brandtii* Radde, 1861, and *L*. *fuscus* Buchner, 1889 [1]. However, molecular data have indicated a kinship between *Lasiopodomys* and *Stenocranius* [2–5], while *L*. *fuscus* has been considered part of the large clade of East Asian voles of genus *Neodon* Hodgson, 1849 [6]. Based on these findings Pavlinov and Lissovsky [7] subdivided *Lasiopodomys* into two subgenera with three species: subgenus *Lasiopodomys* (*L*. *mandarinus* and *L*. *brandtii*) and subgenus *Stenocranius* (*L*. *gregalis* Pallas, 1779). Recently, it has been shown that *L*. *gregalis* is represented by two species–*L*. *gregalis* and *L*. *raddei* Poljakov, 1881 [8,9]. According to the analysis of nuclear genes the genus *Lasiopodomys* originated within the tribe Arvicolini Gray, 1821, approximately 2.4 million years ago (MYA), and the division of that genus into subgenera *Stenocranius* and *Lasiopodomys* occurred about 1.8 MYA [3,10].

Here we aim to test the validity of the subgeneric division of *Lasiopodomys* through cross-species chromosome painting and to define cytogenetic signatures (fusions, fissions, chromosomal association) for it as well as for separation of the *Lasiopodomys* genus from other arvicolins. Previously we described the karyotype of *L*. *gregalis* from the eastern border of the species range and established its chromosomal homology with painting probes of the field vole (*Microtus agrestis* Linnaeus, 1761) [11], but other *Lasiopodomys* species have not been studied by comparative chromosome painting.

The *Lasiopodomys* species are notable for their high karyotype variation. Two species, *L*. *brandtii* and *L*. *gregalis*, have stable diploid chromosome numbers of 34 and 36, respectively, and constant chromosome morphology, in spite of significant morphological variations in *L*. *gregalis* from different populations [3,10,12–15]. The diploid chromosome number of *L*. *mandarinus* varies between 47 and 52 in different populations: 2n = 47–48, NF = 53–55 in Mongolia and Buryatia [16] and 2n = 47–52, NF = 53–55 in China [17–19]. Besides the variation in diploid chromosome number, polymorphism in chromosome morphology often involving two pairs of autosomes (№1 and №2) and the sex chromosomes was described for *L*. *mandarinus* [17–23]. Moreover, *L*. *mandarinus* is one of the few rodent species documented to have an unusual sex chromosome system [17–23]. Three types of sex chromosome systems (XX, XO, XY) with heteromorphic X chromosomes were described for the species based on C- and G-banding comparisons [23].

The current hypothesis on the origin of the unusual sex chromosomes morphology in *L*. *mandarinus*, which suggests that it is the result of translocation of autosomes onto sex chromosomes, was introduced by Wang *et al*. [17]. This hypothesis received support from the analysis of synaptonemal complexes in the Mandarin vole, establishing that the sex chromosomes of *L*. *mandarinus* (XY) pair and recombine at pachytene. It should be noted that the sex chromosomes of *L*. *gregalis* and *L*. *brandtii* do not synapse or recombine during meiosis [24]. We set out to test the autosome to sex chromosome translocation hypothesis of the complex sex chromosome origin in *L*. *mandarinus* through the use of molecular cytogenetic tools. We also used two sets of painting probes, from the field vole (*Microtus agrestis*) and the Arctic lemming (*Dicrostonyx torquatus* Pallas, 1778) [25,26], to track evolutionary chromosome rearrangements in karyotypes of the species *L*. *mandarinus* (LMAN), *L*. *brandtii* (LBRA), and *L*. *gregalis* (LGRE).

## Materials and Methods

### Ethics statement

All experiments were done in accordance with the European Community Council Directive of 22 September 2010 (2010/63/EU) and approved by the Committee on the Ethics of Animal Experiments of the Institute of Molecular and Cellular Biology (IMCB) SB RAS, Russia, and the Committee on the BioEthics of the Zoological Institute, RAS, Saint Petersburg, Russia.

### Species sampled

One female *L*. *mandarinus*, one male *L*. *brandtii*, and one female *L*. *gregalis* specimens used in this study were obtained from the laboratory colonies housed at the Leningrad Zoo and the Institute of Zoology of Russian Academy of Sciences (*L*. *mandarinus* originated from Selenginsky District, Buryatia; *L*. *brandtii*–from Cape Telly, Torey Lake, Borzinskiy District, Chita region, Zabaikalsky Kray; *L*. *gregalis*–from River Kadala, Chitinsky District, Zabaikalsky Kray).

The voles were sacrificed by cervical dislocation. Fibroblast cell lines from skin, lung, and tail biopsies and chromosome suspensions were obtained in the Laboratory of Animal Cytogenetics, the IMCB SB RAS, Russia. Primary fibroblast cell lines were established using enzymatic treatment of tissues as described previously [27,28]. All cell lines were deposited in the IMCB SB RAS cell bank (“The general collection of cell cultures”, № 0310-2016-0002).

### Chromosome preparation and chromosome staining

Metaphase chromosome spreads were prepared from chromosome suspensions obtained from early passages of primary fibroblast cultures as described previously [29–31].

C-banding followed the method of [32] with some modifications [33]. Briefly, the slides were treated with 0.2 N HCl for 20 minutes at room temperature, rinsed in distilled water, and incubated in a freshly prepared 4.6% solution of Ba(OH)_2_ at 53°C for 2–4 min. After thorough rinsing in 0.2 N HCl and several changes of distilled water, the slides were incubated for 1 h at 60°C in 2X SSC, rinsed briefly with distilled water and stained with 2% Giemsa for 7 min.

G-banding was performed on chromosomes of all three species prior to FISH using the standard trypsin/Giemsa treatment procedure [34].

### Fluorescence in situ hybridization (FISH)

The field vole and Arctic lemming painting probes were generated in the Cambridge Resource Centre for Comparative Genomics by DOP-PCR amplification of flow sorted chromosomes and labeled with either biotin or digoxigenin by DOP-PCR amplification as described previously [25,26,35,36]. The telomeric DNA probe was generated by PCR using the oligonucleotides (TTAGGG)_5_ and (CCCTAA)_5_ [37]. Clones of human ribosomal DNA containing the complete 18S-rRNA and 28S-rRNA genes were obtained as described in [38]. FISH was performed following previously published protocols [30,31]. Images were captured using VideoTest-FISH software (Imicrotec) with a JenOptic CCD camera mounted on an Olympus BX53 microscope. Hybridization signals were assigned to specific chromosome regions defined by G-banding patterns previously photographed and captured by the CCD camera. All images were processed using Corel Paint Shop Pro X2 (Jasc Software).

## Results

**Fig 1:**
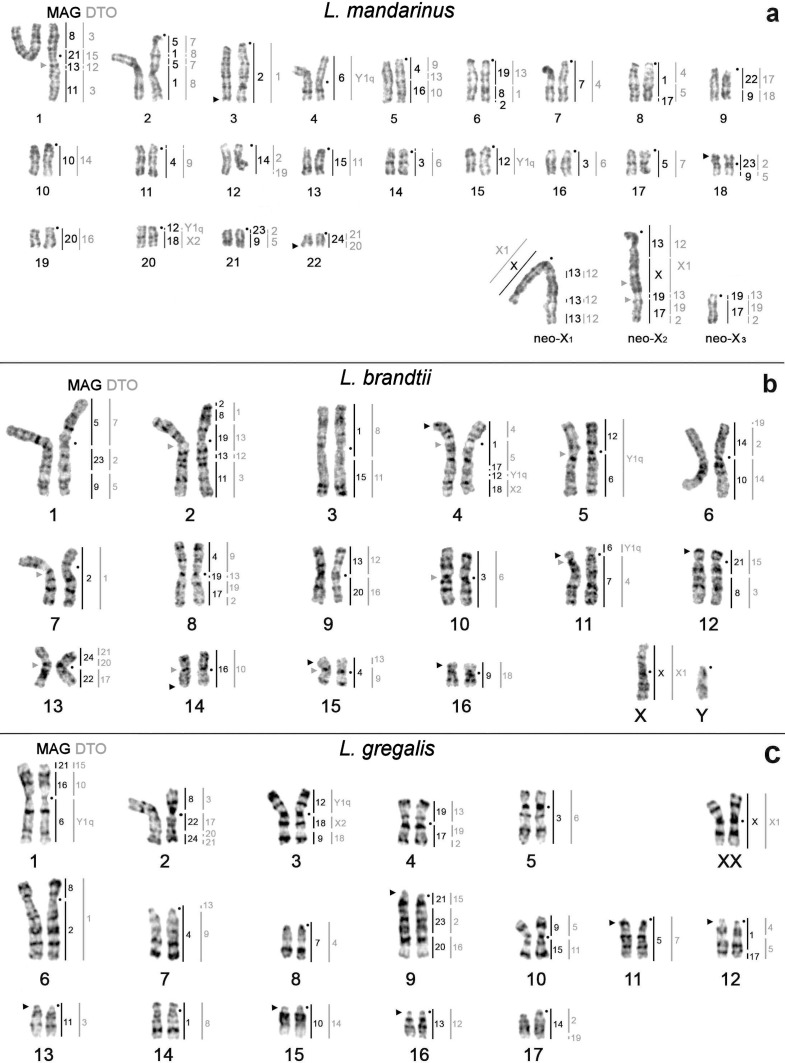
GTG-banded karyotypes of studied species. a–*L*. *mandarinus*, b–*L*. *brandtii*, c–*L*. *gregalis*. Black dots mark the position of centromeres. Vertical black bars mark the localization of *M*. *agrestis* (MAG) chromosome painting probes, while vertical grey bars mark the localization of *D*. *torquatus* (DTO) painting probes. Numbers along the vertical lines correspond to chromosome numbers of *M*. *agrestis* and *D*. *torquatus*. Black triangles indicate sites of localization of rDNA clusters; grey triangles indicate localization of the largest interstitial telomeric blocks.

**Fig 2:**
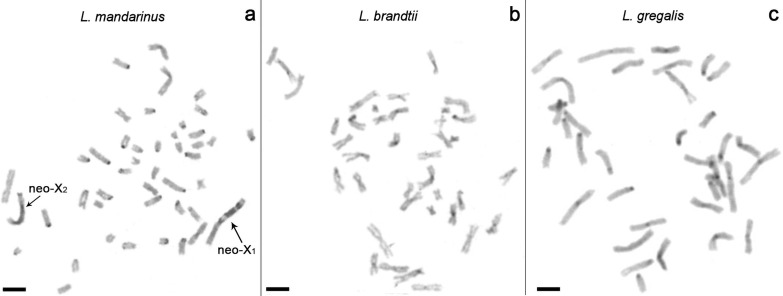
C-banding. a–*L*. *mandarinus*, b–*L*. *brandtii*, с –*L*. *gregalis*. Scale bar is 10 μm.

The male of *L*. *brandtii* and female of *L*. *gregalis* have 2n = 34, NF = 67 and 2n = 36, NF = 54, respectively. The investigated female of *L*. *mandarinus* has 2n = 47 and NF = 55 with 22 pair of autosomes, one metacentric neo-X_1_ chromosome, one submetacentric neo-X_2_ chromosome and one small acrocentric neo-X_3_ (Fig 1A). The metacentric neo-X_1_ chromosome has three large C-positive blocks on the q-arm (Fig 2A).

The field vole and the Arctic lemming painting probes were used to establish chromosomal homologies in the Mandarin vole, Brandt’s vole, and narrow-headed vole (Fig 1). The 24 *M*. *agrestis* autosomal chromosome probes revealed 39 and 34 conserved segments in *L*. *mandarinus* and *L*. *brandtii* karyotypes, respectively (Fig 1A and 1B and S1 Table). In the *L*. *mandarinus* karyotype the MAGX (*Microtus agrestis* X chromosome) probe hybridizes to the p-arm of the metacentric neo-X_1_ chromosomes and to the interstitial part of the q-arm of the submetacentric neo-X_2_ chromosome. MAG13 paints parts of the neo-X_1_ and neo-X_2_ chromosomes, while MAG17 and MAG19 probes hybridize to the distal q-arm of neo-X_2_ and paint the whole neo-X_3_ of *L*. *mandarinus* (Fig 1A).

**Fig 3:**
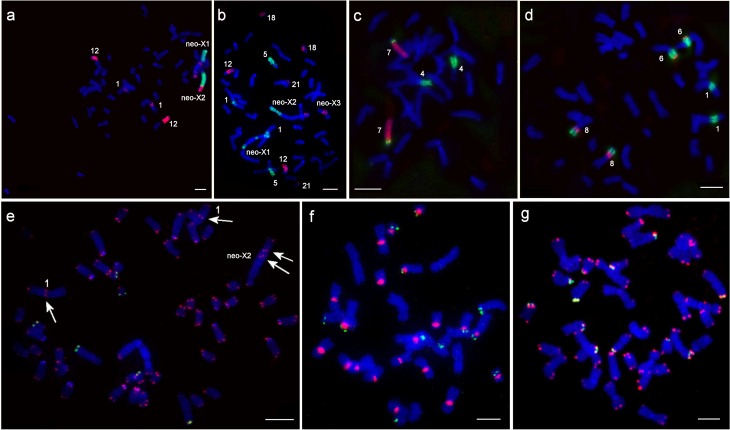
Examples of fluorescence *in situ* hybridization. a–MAGX (green) and MAG13-14 (red) onto *L*. *mandarinus* chromosomes, b–DTO10-12 (green) and DTO2 (red) onto *L*. *mandarinus* chromosomes, c–DTO13 (green) and DTO9 (red) onto *L*. *gregalis* chromosomes, d–DTO2 (green) and DTO19 (red) onto *L*. *brandtii* chromosomes. Examples of fluorescence *in situ* hybridization of the 18S/28S-rDNA probe (green) and telomeric DNA probe (red): e–*L*. *mandarinus* (white arrows indicate localization of the largest interstitial telomeric blocks), f–*L*. *brandtii*, g–*L*. *gregalis*. Scale bar is 10 μm.

The 23 *D*. *torquatus* probes reveal 42, 36 and 32 conserved segments in *L*. *mandarinus*, *L*. *brandtii* and *L*. *gregalis* karyotypes, respectively (Fig 1A and 1C and S1 Table). In the *L*. *mandarinus* karyotype the DTOX1 probe hybridizes to the p-arm of the metacentric neo-X_1_ chromosomes and a middle part of the q-arm of the submetacentric neo-X_2_ chromosome (Fig 1A). Four probes of DTO (2, 12, 13, and 19) hybridize to the heteromorphic pair of sex chromosomes (Fig 1A). Examples of fluorescence *in situ* hybridizations are shown in Fig 3.

The telomeric DNA probe was localized onto chromosomes of all studied species (Fig 3E–3G). Interstitial Telomeric Sequences (ITS) were detected in *L*. *mandarinus* and *L*. *brandtii* (Fig 1A and 1C). In the *L*. *brandtii* karyotype telomeric signals are much weaker than pericentromeric ones distributed on chromosomes 2, 4, 5, 7, 10, 11, 13, 14, 15, and 16 (Fig 3F). In *L*. *mandarinus* non-centromeric ITSs were localized on chromosome 1 and on the neo-X_2_ chromosome (Fig 3E).

We detected six rDNA clusters in *L*. *brandtii* (on chromosomes 4, 11, 12, 14, 15, and 16) and *L*. *gregalis* (on chromosomes 9, 11, 12, 13, 15, and 16) and three rDNA clusters in *L*. *mandarinus* (on chromosomes 3, 18, and 22) (Fig 3E–3G). In *L*. *brandtii* and *L*. *gregalis*, the ribosomal DNA was located at chromosomal segments homologous to MAG1/17 and MAG21 (in associations MAG1/17/12/18 (LBRA4), MAG8/21 (LBRA12) in *L*. *brandtii* and MAG1/17 (LGRE12), MAG20/23/21 (LGRE9) in *L*. *gregalis*).

## Discussion

Unclear taxonomic status of the *Lasiopodomys* and its species composition as well as the unusual structure of sex chromosomes described in *L*. *mandarinus* make this rodent genus especially interesting for cytogenetic studies. In spite of being actively involved in molecular phylogenetic investigations, voles have remained out of deep chromosomal studies for a long time. Here we used cytogenetic tools to test the validity of the subgeneric division of *Lasiopodomys* and its phylogenetic relationships to other Arvicolinae. The use of cross-species chromosome painting allowed us to track evolutionary chromosome rearrangements in karyotypes of three *Lasiopodomys* species, and to support not only the autosome to sex chromosome translocation hypothesis in *L*. *mandarinus* but also to describe the sex chromosomal system in detail.

### The complex sex chromosome system in *L*. *mandarinus*

A particular combination of sex chromosomes usually determines whether an individual is male or female. Most mammalian species have a conventional XX/XY sex chromosome system with the Y-borne testis determining SRY gene. It was proposed that sex chromosomes originated from a pair of autosomes where one of the homologs acquired the sex determining locus [39,40]. Further evolutionary processes led to the accumulation of sex-specific genes on both X and Y chromosomes, suppression of recombination between them and rapid degeneration of Y, occasionally resulting in a complete loss of the Y-chromosome [39]. This process can be further complicated by interchromosomal rearrangements (fusions, fissions, translocations), which result in the emergence of either neo-sex chromosomes or multiple sex chromosome complexes. Neo-sex chromosomes originate from the addition of autosomes or autosomal segments onto original sex chromosomes [41] and in some cases subsequent fission or new fusions of such neo-sex chromosomes could lead to emergence of multiple sex chromosomes [42]. Autosomal material is often added to one of the sex chromosomes or to both of them [36,43,44]. Even in cases with a known composition of the neo-sex chromosome complexes, many questions remain unanswered regarding behavior of those elements in meiosis, localization of sex-determining loci and the role of neo-chromosome complexes in speciation etc.

Although some sex chromosome systems other than XX/XY are found within various mammalian taxa, rodents demonstrate the widest range of sex chromosome systems and even mechanisms of sex determination (see [45] and references therein). Many species with unusual sex chromosome systems belong to the Arvicolinae subfamily, and the Mandarin vole is one of them. Polymorphism in the sex chromosomes of *L*. *mandarinus* has been described previously based on G- and C-banding analysis only [16–19,22]. Wang *et al*. proposed that such a polymorphism might be caused by the translocation of autosomes to the X chromosome [17].

Here the application of chromosome painting provides evidence for the origin of neo-sex chromosomes in *L*. *mandarinus* by at least two independent autosome-sex chromosome translocation events. The complex of sex chromosomes in the *L*. *mandarinus* female described here consists of one metacentric chromosome (neo-X_1_), one submetacentric chromosome (neo-X_2_) and one small acrocentric referred to here as “neo-X_3_^”^. At least two pairs of ancestral autosomes participated in the formation of these neo-X chromosomes. We propose the following description of the karyotype of the female Mandarin vole: 47, neo-X_1_, neo-X_2_, neo-X_3_, where “47” is the diploid number and “neo-X” is a description of sex chromosomes.

This finding is consistent with the theory proposed based on the unusual synapsis in meiosis of the Mandarin vole [24]. The XY bivalent of *L*. *mandarinus* contains two pairing regions. Both pairing regions are relatively long and both take part in regular recombination, similar to the human pseudoautosomal regions [24]. These regions may have originated by *de novo* translocation of autosomal segments, as proposed for the human pairing regions [46].

The formation of the metacentric neo-X_1_ was accompanied by heterochromatin accumulation in the part of the X chromosome homologous to MAG13. It seems likely that either the ancestral X chromosome or the ancestral autosomal segment carried a heterochromatic block that prevented the spread of chromosome inactivation into the autosomal compartment after the translocation [47]. We conjecture that a fusion of MAG13/X with MAG17/19 took place relatively recently as the region of fusion retained ITS sites (Figs 1A and 3E–3G). Thus we have characterized the complex chromosomal composition of the sex trivalent in the female Mandarin vole. Nevertheless, to understand this phenomenon at the molecular and sequence level further studies are required, involving additional individuals from both sexes and an in-depth study of the localization and function of sex-determining genes.

### Cytogenetic data support a close relationship between subgenus *Lasiopodomys* and subgenus *Stenocranius*

Recently there has been much discussion on taxonomic revision of the genus *Lasiopodomys* concerning the status of the whole taxon, its species composition, and in particular the division of the genus into subgenera *Stenocranius* (*L*. *gregalis* and *L*. *raddei*) and *Lasiopodomys* (*L*. *mandarinus* and *L*. *brandtii*) [2–5,8,9]. Often, especially when morphologically similar species are studied, cytogenetic characters provide additional phylogenetic data for possible problem-solving.

The presence of MAG1bc/17b (an association common for almost all Arvicolinae and containing the fragment MAG1b/17b which was also found in some Cricetinae species) and MAG2/8a (common for all Arvicolinae) in karyotypes of the three studied *Lasiopodomys* species may be considered as a synapomorphic trait of the Arvicolinae subfamily. MAG12/18, 17a/19, and 22/24 associations have not been found previously in karyotypes of other rodents and may be the defining markers for the genus *Lasiopodomys* (Table 1). It is important to stress that *L*. *brandtii* and *L*. *gregalis* carry rDNA clusters at chromosomal segments homologous to MAG1bc/17b, probably sharing the same origin (the same for rDNA clusters in *L*. *brandtii* and *L*. *gregalis* located at chromosomal segments homologous to MAG21).

**Table 1:** Distribution of shared syntenic segment associations.

Association of MAG chromosomes	Association of DTO chromosomes	LMAN	LBRA	LGRE	Presence of association in other taxonomic groups	Reference
1b/17b	5	+	+	+	common for all Arvicolinae and some Cricetinae species	[25]
1bc/17b	4/5	+	+	+	all Arvicolinae except *Dicrostonyx torquatus* and *Ellobius talpinus*	[25]
2/8a	1	+	+	+	all Arvicolinae	
8/19	3/13				*Microtus dogramacii*; *Alexandromys maximowiczii*	[11]
8/19	1/13	+	+			
2a/8a/19b	1/13	+	+			
8b/21	3/15	+	+			
9b/23	5/2	+	+		*Ellobius talpinus*	[48]
9/23	18/2				*Mesocricetus auratus*	[25]
11/13b	3/12a	+	+		*Arvicola amphibius*	[25]
11/13	3/12				*Arvicola amphibius*	[25]
12b/18	Y1qa/X2	+	+	+		
12/18	Y1q/X2			+		
17a/19a	2/19/13a	+	+	+		
17a/19	2/19/13b			+		
22/24	17/20/21		+	+		

MAG–*M*. *agrestis*, DTO–*D*. *torquatus*, LMAN–*L*. *mandarinus*, LBRA–*L*. *brandtii*, LGRE–*L*. *gregalis*.

The associations MAG2a/8a/19b, 8b/21, 9b/23, 11/13b, 12b/18, and 17a/19a were found in the karyotypes of *L*. *mandarinus* and *L*. *brandtii* and could be characteristic for the subgenus *Lasiopodomys*. Although some of these associations seem to have a shared origin with other arvicolin or cricetin species, the use of Arctic lemming painting probes turned out to be instrumental in resolving and disproving these seemingly shared associations (Table 1).

Despite the fact that MAG12/18 and MAG17a/19 are the key associations synapomorphic for the whole genus *Lasiopodomys*, the fissions of MAG12 and MAG19 leading to the formation of MAG12a, MAG12b/18 and MAG17a/19a, MAG19b (included in MAG2a/8a/19b), respectively, are characteristic only for the subgenus *Lasiopodomys*. The karyotype of *L*. *mandarinus* is characterized by a high fragmentation in comparison with the karyotypes of its sister species. The absence of the association MAG22/24 (= DTO17/20/21) in the *L*. *mandarinus* karyotype was probably a secondary event due to chromosome fission. However, we cannot exclude the possibility that the presence of the association in karyotypes of *L*. *brandtii* and *L*. *gregalis* only was a result of convergent evolution. In this case *L*. *mandarinus* retained the ancestral condition. Thus, the origin of the association MAG22/24 has not been unambiguously defined.

The shared chromosomal associations found here seem to be in agreement with the evolutionary closeness of *L*. *mandarinus* and *L*. *brandtii* and their isolation from *L*. *gregalis*, providing cytogenetic evidence for the taxonomic division of subgenera based on DNA sequence analyses [2–5]. Moreover, the cytogenetic data reveal possible chromosomal signatures (12/18, 17a/19, and 22/24) that support the separate position of the genus inside Arvicolinae.

### Rapid karyotype evolution in *Lasiopodomys*

The number of chromosomal rearrangements occurring in a certain evolutionary period indicates the rate of evolutionary karyotype reorganization. Previously it was revealed that the rate of karyotype evolution varies greatly (in several folds) across the mammalian phylogenetic tree [49]. In rodents the average rate of karyotype evolution has been determined as one fusion/fission per MY [50]. Arvicolinae belongs to the group of species characterized by an even higher tempo of chromosomal reorganization [11].

**Fig 4:**
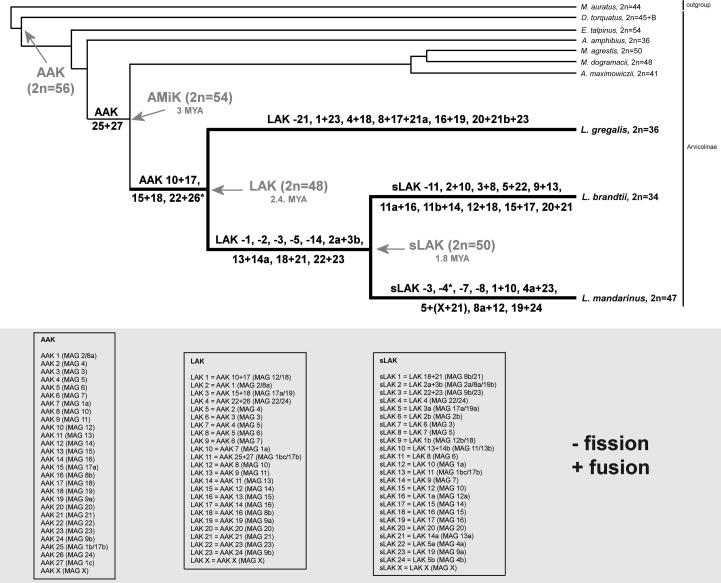
Karyotype evolution pathways in three *Lasiopodomys* species. Tree topology is based on the molecular phylogeny of Arvicolinae species presented by [3]. AAK–ancestral Arvicolinae karyotype, AMiK–ancestral karyotype of the tribe Arvicolini, LAK–ancestral karyotype of the genus *Lasiopodomys*, sLAK–ancestral karyotype of the subgenus *Lasiopodomys*. Chromosome numbers are indicated in AAK, LAK, and sLAK segments. *–see Discussion.

The cladistic analysis of chromosomal rearrangements did not provide a well-resolved tree with valid support for arvicolin genera branching due to the shortage of phylogenetically informative chromosomal characters (our unpublished data). However, the availability of the molecular phylogeny of Arvicolinae species [3], a previously proposed ancestral karyotype for the subfamily Arvicolinae (Ancestral Arvicolinae Karyotype, AAK) [25], an ancestral karyotype for the tribe Arvicolini (AMiK) [11], and cytogenetic data on *Lasiopodomys* species-specific characters allow us to track chromosomal exchanges and calculate the tempo using molecular estimates of divergence times (Fig 4).

Most phylogenetic studies support the early branching of the common ancestor of the genus *Lasiopodomys* from the stem lineage of tribe Arvicolini [3,7,10]. We revealed only one cytogenetic character which could support the basal position of *Lasiopodomys* in the tribe Arvicolini: karyotypes of all studied species of the genus *Lasiopodomys* have two fragments of MAG1 (see S1 Table), with the exception of the *L*. *mandarinus* karyotype in which three segments of MAG1 (LMAN2) were detected as a result of a secondary inversion. Thus, the two-segment state of MAG1 links the genus *Lasiopodomys* and tribe Arvicolini, whereas the possible presence of three segments homologous to MAG1 was shown to be part of AAK [25].

The presumptive ancestral karyotype of the whole genus *Lasiopodomys* (LAK) could be formed from AMiK by three Robertsonian fusions (Fig 4 and S1 Fig). According to phylogenetic estimates the basal radiation of genera *Dicrostonyx*, *Prometheomys*, *Ondatra* and tribe Lemmini took place 6.5, 6.8, 7.7 and 7.2 MYA, respectively, and the divergence time of Arvicolinae/Cricetinae was calculated to be 18.1 MYA [3]. Based on these data we can assume that AMiK was formed about 3 MYA and LAK–about 2.4 MYA. So the rate of chromosomal rearrangements in the branch leading from AAK to LAK is about one rearrangement per 3 million years (MY). However, the genus *Lasiopodomys* demonstrates an increased rate of chromosomal rearrangements in comparison with the most other rodents: the karyotype of *L*. *gregalis* evolved from the presumptive LAK by one fission and seven fusions, roughly four chromosomal rearrangements per MY.

The putative common ancestral karyotype for the subgenus *Lasiopodomys* (sLAK) could have been formed from LAK by five fissions and four fusions (Fig 4 and S1 Fig); the karyotype of *L*. *brandtii* could be produced from sLAK mainly by Robertsonian fusions (9 fusions) and one fission. Interestingly, the karyotype of *L*. *mandarinus* was shaped by a number of complex fissions, fusions and inversions (Fig 4 and S2 Fig). In total, both species have undergone around 20 chromosomal rearrangements in 2.4 MYA [3]. So, the rate of chromosomal rearrangements was increased in subgenus *Lasiopodomys* up to eight chromosomal rearrangements per MY.

So the rate of chromosomal exchanges steadily increased during formation and radiation of the *Lasiopodomys* species complex. Interestingly, we observed that the number of ribosomal DNA clusters has the opposite trend and is much lower in currently evolving *L*. *mandarinus* (three) than in constant karyotypes of *L*. *brandtii* and *L*. *gregalis* (six) (Figs 1 and 3E–3G). Previously, it was hypothesized using the ground vole as an example that the “primitive” (ancestral) karyotype is defined by the high number of NORs, whereas species with extensively rearranged karyotypes have a lower number of NORs [51]. Although we have previously shown the hypothesis of multiple NORs as a characteristic of the primitive state this does not hold in other species of voles [25]. Perhaps in this case, the few rDNA clusters in the Mandarin vole was a consequence of the extensive chromosomal rearrangements that shaped the species karyotype.

Interestingly, the increased rate of chromosome reshuffling in *Lasiopodomys* is not the only sign of fast evolutionary genomic changes. An extremely high mutation rate in the mitochondrial cytochrome b gene was shown in *L*. *gregalis* during a previous species-wide phylogeographic study. The estimated rate is an order of magnitude higher than previous estimates for *Microtus* species. A high genetic diversity was revealed both among and within the narrow-headed vole mtDNA lineages [3,10].

## Conclusion

Our molecular cytogenetic analyses have discovered chromosomal associations shared by *L*. *gregalis* with *L*. *mandarinus* and *L*. *brandtii*, which unite the three species and support the monophyly of *Lasiopodomys*. The karyotypes of *Lasiopodomys* have evolved through a complicated chain of reshuffling events involving Robertsonian fusions, chromosomal fissions, inversions and heterochromatin expansion. The findings also provide strong support for the previously suggested subgeneric division of genus *Lasiopodomys*: the subgenus *Stenocranius* is separated from subgenus *Lasiopodomys* by eight rearrangements that occurred within a short evolutionary period, less than 1.8 MY [3,10]. The fast tempo of chromosome evolution involved not only autosomes, but also sex chromosomes, forming an unusual complex of sex chromosomes in at least one specimen of the Mandarin vole, with an elaborate combination of expanded heterochromatin and autosomal/sex chromosomal rearrangements. We provide molecular cytogenetic evidence that validates the hypothesis of autosomal translocation to sex chromosomes [17] and explains the heteromorphism of the X chromosomes in *L*. *mandarinus*. We further hypothesize that the *L*. *mandarinus* karyotype originated over a short evolutionary period (less than 1.8 MY) by extensive genomic rearrangements: 10 fusions/fissions, rDNA location, ITS, heterochromatin expansion, and sex chromosomes rearrangements. We assume that these processes might be still ongoing in the current populations. It remains to be answered why karyotypes of *L*. *gregalis* and *L*. *brandti* that were formed as result of fast chromosome evolution do not exhibit signs of cytogenetic variation in current populations. Thus, cytogenetic studies of larger number of individuals from the *Lasiopodomys* genus could provide more clues as to the role of chromosome rearrangement in complex speciation events.

## Supporting Information

S1 Fig A hypothetical path of formation of the presumed ancestor karyotype of the genus *Lasiopodomys* (LAK) from the ancestral karyotype of the tribe Arvicolini (AMiK) [11] and a hypothetical path of formation of the presumed ancestral karyotype of *L*. *mandarinus* and *L*. *brandtii* (sLAK) from LAK (ancestral *Lasiopodomys* karyotype). Single chromosomes of *M*. *agrestis* represented by one element in AAK and LAK are shown in blue. The chromosomes represented by two elements are marked by pairs of various colors. Numbers along the segments correspond to chromosome numbers of *M*. *agrestis*. Plus signs indicate chromosome fusions. *–see Discussion.
(TIF)

S2 Fig A hypothetical path of evolutionary rearrangements of key chromosomes leading to *L*. *mandarinus* karyotype formation from sLAK. Numbers along the chromosomes correspond to chromosome numbers of *M*. *agrestis*. Minus signs indicate chromosome fissions, plus signs indicate chromosome fusions. *–see Discussion.
(TIF)

S1 Table Number of chromosomal segments painted by isolated *M*. *agrestis* and *D*. *torquatus* chromosomes in individual voles of *L*. *mandarinus*, *L*. *brandtii* and *L*. *gregalis* studied here. (DOC)

## Abbreviations

2n: diploid number of chromosomes
AAK: ancestral Arvicolinae karyotype
AMiK: ancestral karyotype of the genus *Microtus*
DTO: *Dicrostonyx torquatus*
FISH: fluorescence *in situ* hybridization
GTG-banding: G-banding by trypsin using Giemsa
ITS: interstitial telomeric sequences
LAK: ancestral karyotype of the genus *Lasiopodomys*
LBRA: *Lasiopodomys brandtii*
LGRE: *Lasiopodomys gregalis*
LMAN: *Lasiopodomys mandarinus*
MAG: *Microtus agrestis*
MYA: million years ago
MY: million years
NFa: fundamental autosomal number
sLAK: ancestral karyotype of the subgenus *Lasiopodomys*
