# Supplementary figures and images for: Rapid Karyotype Evolution in Lasiopodomys Involved at Least Two Autosome – Sex Chromosome Translocations
Source: PLoS One. 2016 Dec 9;11(12):e0167653. doi: 10.1371/journal.pone.0167653 (PMC5147937; doi:10.1371/journal.pone.0167653)

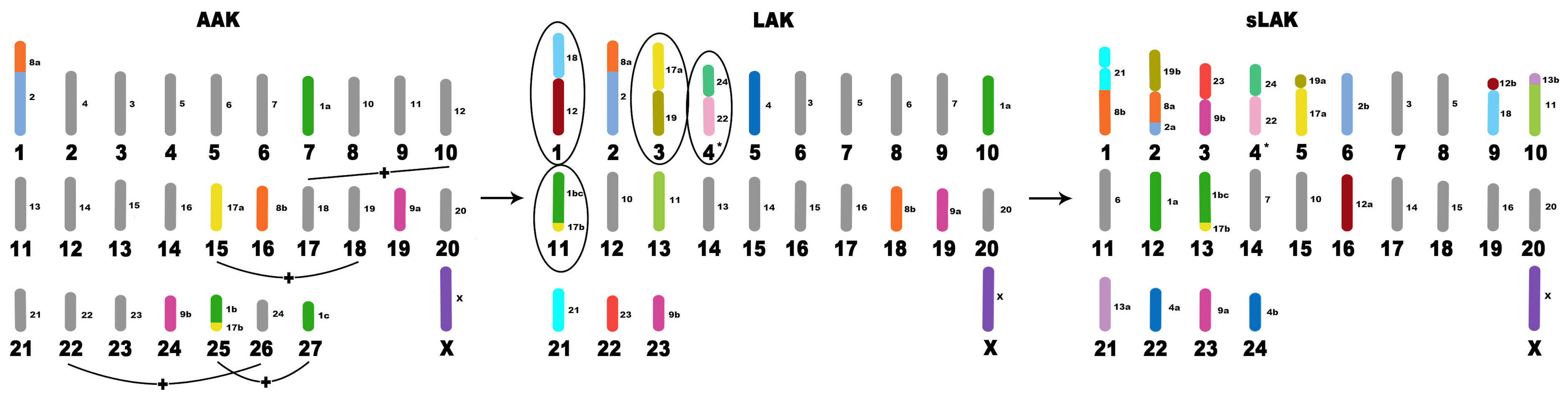

Supplement: S1 Fig — Single chromosomes of M. agrestis represented by one element in AAK and LAK are shown in blue. The chromosomes represented by two elements are marked by pairs of various colors. Numbers along the segments correspond to chromosome numbers of M. agrestis. Plus signs indicate chromosome fusions. *–see Discussion. (TIF) [file pone.0167653.s001.tif]

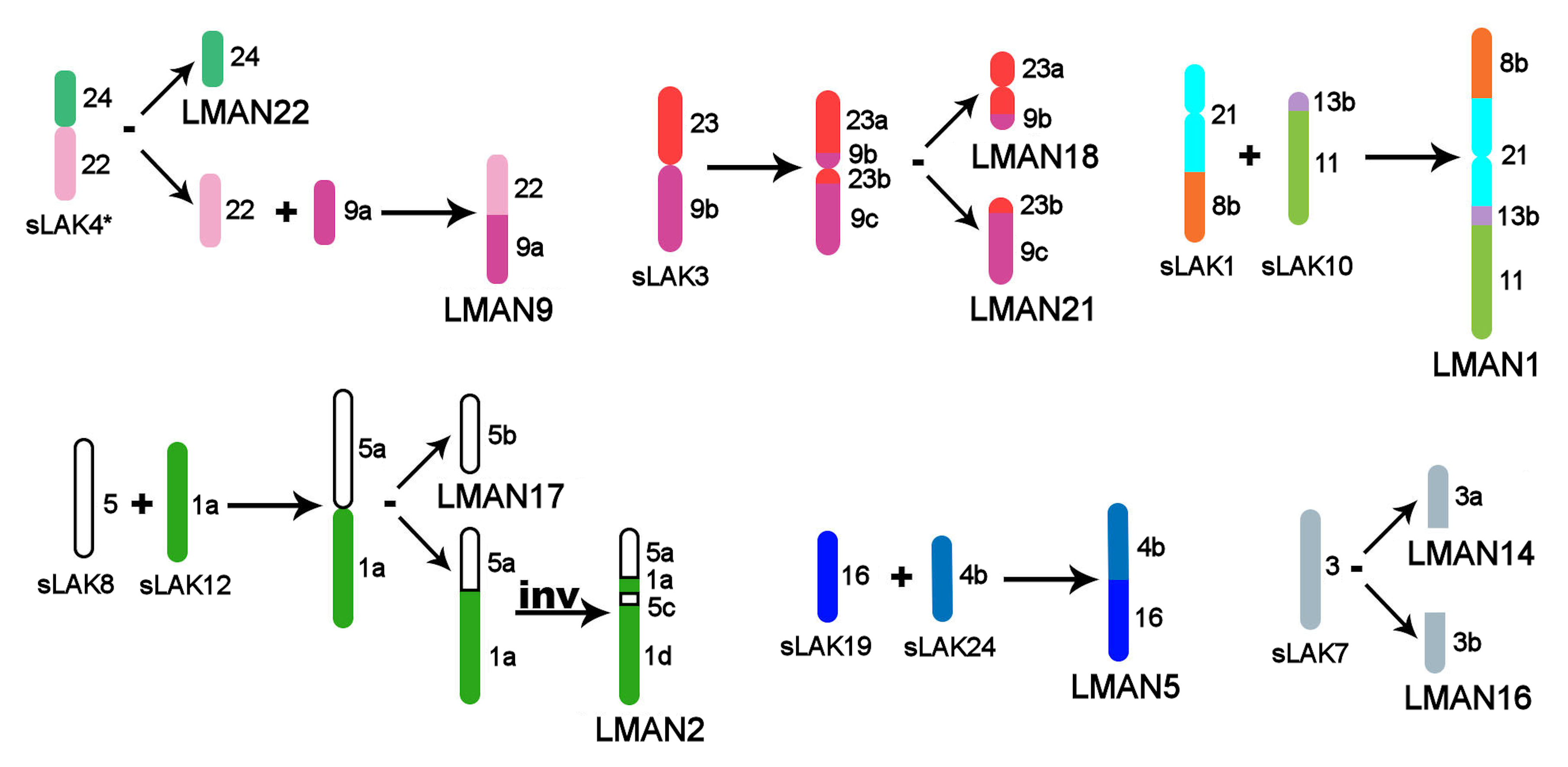

Supplement: S2 Fig — Numbers along the chromosomes correspond to chromosome numbers of M. agrestis. Minus signs indicate chromosome fissions, plus signs indicate chromosome fusions. *–see Discussion. (TIF) [file pone.0167653.s002.tif]
