# Supplementary material for: Rapid Karyotype Evolution in Lasiopodomys Involved at Least Two Autosome – Sex Chromosome Translocations
Source: PLoS One. 2016 Dec 9;11(12):e0167653. doi: 10.1371/journal.pone.0167653 (PMC5147937; doi:10.1371/journal.pone.0167653)
Supplement: S1 Table — (DOC) [file pone.0167653.s003.doc]

| **Chromosomes of *M. agrestis*** | **LMAN** | **LBRA** | **LGRE^*^** | **Chromosomes of *D. torquatus*** | **LMAN** | **LBRA** | **LGRE** |
| --- | --- | --- | --- | --- | --- | --- | --- |
| **MAG1** | 3 | 2 | 2 | **DTO1** | 2 | 2 | 1 |
| **MAG2** | 2 | 2 | 1 | **DTO2** | 4 | 3 | 3 |
| **MAG3** | 2 | 1 | 1 | **DTO3** | 2 | 2 | 2 |
| **MAG4** | 2 | 2 | 1 | **DTO4** | 2 | 2 | 2 |
| **MAG5** | 3 | 1 | 1 | **DTO5** | 3 | 2 | 2 |
| **MAG6** | 1 | 2 | 1 | **DTO6** | 2 | 1 | 1 |
| **MAG7** | 1 | 1 | 1 | **DTO7** | 3 | 1 | 1 |
| **MAG8** | 2 | 2 | 2 | **DTO8** | 2 | 1 | 1 |
| **MAG9** | 3 | 2 | 2 | **DTO9** | 2 | 2 | 1 |
| **MAG10** | 1 | 1 | 1 | **DTO10** | 1 | 1 | 1 |
| **MAG11** | 1 | 1 | 1 | **DTO11** | 1 | 1 | 1 |
| **MAG12** | 2 | 2 | 1 | **DTO12** | 2 | 2 | 1 |
| **MAG13** | 2 | 2 | 1 | **DTO13** | 3 | 3 | 2 |
| **MAG14** | 1 | 1 | 1 | **DTO14** | 1 | 1 | 1 |
| **MAG15** | 1 | 1 | 1 | **DTO15** | 1 | 1 | 2 |
| **MAG16** | 1 | 1 | 1 | **DTO16** | 1 | 1 | 1 |
| **MAG17** | 2 | 2 | 2 | **DTO17** | 1 | 1 | 1 |
| **MAG18** | 1 | 1 | 1 | **DTO18** | 1 | 1 | 1 |
| **MAG19** | 2 | 2 | 1 | **DTO19** | 2 | 2 | 2 |
| **MAG20** | 1 | 1 | 1 | **DTO20** | 1 | 1 | 1 |
| **MAG21** | 1 | 1 | 2 | **DTO21** | 1 | 1 | 1 |
| **MAG22** | 1 | 1 | 1 | **DTOY1q** | 3 | 3 | 2 |
| **MAG23** | 2 | 1 | 1 | **DTOX2** | 1 | 1 | 1 |
| **MAG24** | 1 | 1 | 1 |  |  |  |  |
| **Total number of conserved autosomal segments** | 39 | 34 | 29 | **Total number of conserved autosomal segments** | 42 | 36 | 32 |

**^*^** Localization of painting probes of the field vole on *L. gregalis* chromosomes shown previously in [11].
